# Do Cross-Language Script Differences Enable Bilinguals to Function Selectively When Speaking in One Language Alone?

**DOI:** 10.3389/fcomm.2021.668381

**Published:** 2021-06-22

**Authors:** Noriko Hoshino, Anne L. Beatty-Martínez, Christian A. Navarro-Torres, Judith F. Kroll

**Affiliations:** 1 Department of English, Tsuda University, Kodaira, Japan; 2 Department of Psychology, McGill University, Montréal, QC, Canada; 3 Department of Language Science, University of California, Irvine, Irvine, CA, United States

**Keywords:** different-script bilinguals, cross-language activation, semantic interference, phono-translation, picture-word interference task

## Abstract

The present study examined the role of script in bilingual speech planning by comparing the performance of same and different-script bilinguals. Spanish-English bilinguals (Experiment 1) and Japanese-English bilinguals (Experiment 2) performed a picture-word interference task in which they were asked to name a picture of an object in English, their second language, while ignoring a visual distractor word in Spanish or Japanese, their first language. Results replicated the general pattern seen in previous bilingual picture-word interference studies for the same-script, Spanish-English bilinguals but not for the different-script, Japanese-English bilinguals. Both groups showed translation facilitation, whereas only Spanish-English bilinguals demonstrated semantic interference, phonological facilitation, and phono-translation facilitation. These results suggest that when the script of the language not in use is present in the task, bilinguals appear to exploit the perceptual difference as a language cue to direct lexical access to the intended language earlier in the process of speech planning.

## INTRODUCTION

Although bilinguals are able to speak each language without apparent intrusion of the other language, experimental studies demonstrate that both languages are active even when utterances are planned in one language alone and that bilinguals eventually select the intended language (see Costa, 2005; Kroll et al., 2006, Kroll et al., 2008, for reviews). A notable feature of much of the research on this topic is that it has examined the performance of bilinguals whose two languages use the same written script and are therefore potentially ambiguous with respect to language status. For speakers of languages like Dutch and English or Spanish and Catalan, there are many words that have similar orthography and phonology. Cross-language ambiguity may extend the process of language selection because ambiguity may increase cross-language competition. The question we ask in the present paper is whether differences in the written script of two languages can effectively reduce activation of the language not in use to allow bilinguals to select the target language, the language of production, earlier in the process of speech planning. If bilinguals can exploit cross-language differences to enable lexical access to be language selective, they may be better able to constrain the scope of competition and reduce functional demands on control processes. We examined this question by comparing the performance of Spanish-English and Japanese-English bilinguals in a picture-word interference task.

Past studies on different-script word production have reported findings that are similar to those for same-script production in that the consequences of cross-language competition appear to be evident (e.g., Hoshino and Kroll, 2008; Guo et al., 2011; Misra et al., 2012; Moon and Jiang, 2012). For example, Hoshino and Kroll (2008) found that both Japanese-English and Spanish-English bilinguals produced cognate facilitation in a simple picture naming task in English, their second language (L2). In simple picture naming, the written script of each language is absent but Hoshino and Kroll hypothesized that a bilingual’s experience with two languages that differ not only in script but in a variety of lexical and syntactic features might also serve as a cue to allow speech planning to be selective. Finding significant cognate facilitation for Japanese-English bilinguals means that the phonology of the Japanese name of the picture was activated when planning to speak the word in English. However, finding phonological activation of the first language (L1) during speech planning in L2 in the absence of the printed word in L1 does not mean that script information is unimportant when it is present; it is only that it does not appear to modulate processing when it is absent.

Although it might seem that the written lexical form should not influence word production, there is a great deal of evidence to suggest that semantics, phonology, and orthography are active to some degree in both comprehension and production (e.g., Tan and Perfetti, 1999; Damian and Bowers, 2003; Ziegler et al., 2003; Chéreau et al., 2007; Bi et al., 2009; Pattamadilok et al., 2009; Zhang et al., 2009; Zhang and Weekes, 2009; Rastle et al., 2011). The effects of orthography on production depend on task demands and have been reported to be reliable only when the written lexical form is overtly present in the task such as form preparation and picture-word interference paradigms (e.g., Roelofs, 2006; Alario et al., 2007; Damian and Bowers, 2009). The resonance across lexical codes suggests that each code may eventually activate whatever information is similar to it both within and across languages. For different-script languages, the implication is that the presence of phonological overlap may be sufficient to observe cross-language interactions. However, the cross-language phonological effect of the sort that Hoshino and Kroll (2008) reported does not mean that different-script bilinguals are unable to exploit language-specific cues when the words are actually present. The current study asks the question of whether different-script bilinguals can exploit script differences when the words are actually present in the task.

Models of lexical access in bilingual word production must address the locus and manner of language selection if the intention to speak one language alone is not sufficient to restrict activation to that language (for reviews, see Costa, 2005; Kroll et al., 2006; Kroll et al., 2008). Although past bilingual research concurs with the view of nonselective activation, particularly in L2 production, there has been debate over the manner of selection. At issue is whether all activated lexical alternatives become candidates for selection. According to language-nonspecific (competition for selection) models, candidates from both languages are active competitors (e.g., Hermans et al., 1998; Kroll et al., 2008; Hoshino and Thierry, 2011). On this view, there may be inhibition of alternatives in the unintended language (e.g., Meuter and Allport, 1999; Philipp and Koch, 2009). In contrast, language-specific models (e.g., Costa et al., 1999) assume that lexical alternatives may be active in both languages but only those in the response language are considered for selection. Kroll et al. (2008) describe this as a “mental firewall” model because the assumption is that activity on the wrong side of the firewall has little consequence for lexical selection. Alternatively, some models assume that there is no competition even within a language in the process of lexical selection. The Response Exclusion Hypothesis posits that the target and non-target alternatives compete at a post-lexical level, not at a lexical level (Mahon et al., 2007).

In theory, cross-language script differences could affect activation and/or selection. If script differences provide categorical cues to language membership, then there might not be any activation of the non-target language or that activation might subside more quickly relative to a same-script language. The results of Hoshino and Kroll (2008) suggest that in the absence of perceptual information that might cue the language of production, there appears to be similar activation of the non-target language for same and different-script bilinguals. Thus, knowledge of the different script alone does not seem to suffice to reduce the activation of cross-language phonology. Alternatively, both languages might be activated but the different script information might better enable selection to occur at an earlier stage of speech planning than otherwise possible. Again, the presence of similar cognate facilitation for both same and different-script bilinguals suggests that different language script itself does not alter the locus of language selection. However, without an explicit test of how the other language script is processed when it is perceptually present, it is premature to conclude that script does not influence cross-language activation or selection.

Debate in the past literature on bilingual language production regarding the locus and manner of selection results in part from the use of different experimental paradigms. Many bilingual production studies used the picture-word interference paradigm in which bilinguals name a picture in one language while ignoring a visually or auditorily presented distractor word in the same or other language (e.g., Hermans et al., 1998; Costa and Caramazza, 1999; Costa et al., 1999; Costa et al., 2003; Knupsky and Amrhein, 2007; Hoshino and Thierry, 2011; Boukadi et al., 2015; Giezen and Emmorey, 2016). In these experiments, the relation between the distractor word and the picture’s name is varied along with the timing of the distractor presentation relative to the picture, and the language of the distractor. The logic of these studies is to examine the time course of distractor effects as a way of identifying the activity of the non-target language during each stage of production. Past research using picture-word interference has reported compelling effects of the language not in use. The evidence on picture-word interference with same-script bilinguals suggests that even when bilinguals are producing words in one language alone and the distractor is in the same language as production, there is momentary activation of the other language (e.g., Hermans et al., 1998; Hoshino and Thierry, 2011). In other words, the non-target language is activated in single language as well as mixed language contexts.

Although most of the past research using the picture-word interference paradigm has examined the performance of bilinguals whose languages share the same Roman alphabets, a few studies have examined the effect of language similarity on cross-language activation and lexical selection. Boukadi et al. (2015) replicated past findings for same-script bilinguals in a group of different-script Arabic-French bilinguals, with the distractor presented auditorily in the non-target language. However, no cross-language effect was observed when the language of the distractor was the same as the target language, the language of production. Similarly, Giezen and Emmorey (2016) showed that hearing bilinguals who were proficient users of English and American Sign Language (ASL), activated English when naming pictures in ASL. One noticeable finding of this study was that unlike past picture-word interference research with same-script bilinguals (e.g., Hermans et al., 1998; Costa et al., 1999; Hoshino and Thierry, 2011) and different-script bilinguals (Boukadi et al., 2015), no semantic interference effect was observed for bimodal bilinguals when they ignored semantically related English words while producing ASL. Giezen and Emmorey (2016) suggested that semantically related English distractor words and target ASL signs do not compete in the post-lexical articulatory buffer or that there might be time course differences between sign production and spoken word production. Although bimodal bilinguals can produce two languages (both signs and words) simultaneously unlike unimodal bilinguals, the control mechanism involved in the process of speech production is more similar than different (e.g., Blanco-Elorrieta et al., 2018; Emmorey et al., 2020a). The important point of these past studies with different-script and bimodal bilinguals is that there was cross-language activation when distractor words were presented auditorily in the non-target language. It is possible that different scripts would modulate cross-language activation if the distractor words were presented visually rather than auditorily.

What evidence exists that visually presented distractors might provide cues to the language of production? Miller and Kroll (2002) reported evidence for the language cue hypothesis in a translation Stroop study with same-script bilinguals. The translation Stroop task is formally similar to picture-word interference but instead of a picture as the initiating event, a word is presented for translation. The task is to translate the word as quickly as possible while ignoring a distractor word. When the distractor word appeared in the language of production (e.g., see a word in Spanish to be translated into English and the distractor word appears in English), Miller and Kroll found semantic interference and form facilitation replicating previous translation and picture-word interference studies (La Heij et al., 1990). However, when the distractor word appeared in the language of the target word to be translated (e.g., see a word in Spanish to be translated into English but the distractor appears in Spanish), they found that there was neither semantic interference nor form facilitation. They argued that in translation, unlike picture naming, there is a cue to language membership available in the target word that initiates speech planning. If the word appears in Spanish in a translation task, the bilingual knows not to speak Spanish. If script differences function as explicit cues to language status and if bilinguals can exploit language-specific information, then the process of planning the spoken utterance becomes similar to a within-language process in which only candidates in the language to be produced compete for selection. Because English and Japanese differ in script, there may be stronger cues for language status than for English and Spanish. Indeed, color Stroop studies with different-script bilinguals and trilinguals have demonstrated that when two or three languages differ in script, bilinguals and trilinguals experience less cross-language interference (e.g., Smith and Kirsner, 1982; Chen and Ho, 1986; Brauer, 1998; Lee and Chan, 2000; Van Heuven et al., 2011).

We note that the results of color Stroop studies are limited given that they include only color names and the number of mappings is small. Therefore, in the present study, we used a picture-word interference paradigm, which allowed us to include a variety of conditions. Similar to past bilingual studies that have used this paradigm, the experiment we report included four types of distractor words in relation to the name of the target picture: phonologically related to the picture name, semantically related to the picture name, phonologically related to the translation of the picture name (phono-translation), and the translation of the picture name. Each of these distractor words was matched with an unrelated control. Same-script Spanish-English bilinguals (Experiment 1) and different-script Japanese-English bilinguals (Experiment 2) were asked to name noncognate pictures in their L2 English while ignoring visually presented distractors in their L1 Spanish or Japanese. In other words, the present study examined picture naming in L2 in the presence of distractors in L1 because those are the conditions that typically produce the largest cross-language effects. For Spanish-English bilinguals, we expected to replicate previously reported results, i.e., phonological facilitation, semantic interference, phono-translation interference, and translation facilitation. On the other hand, if distractor words in a different-script language provide a language cue to production that bilinguals are able to exploit, there should be no effect of distractor conditions when Japanese-English bilinguals produce picture names in English in the presence of Japanese distractor words.

## EXPERIMENT 1: SPANISH-ENGLISH BILINGUALS

### Method

#### Participants

Forty-eight Spanish-English bilinguals participated in Experiment 1. They were all living in the L2 environment at the time of testing. After completing the main picture-word interference task, participants were given a language history questionnaire (Tokowicz et al., 2004), an English lexical decision task (Azuma and Van Orden, 1997), a semantic verbal fluency task (Linck et al., 2009), Simon task (Bialystok et al., 2004; Linck et al., 2008), and an operation span task (Tokowicz et al., 2004) as a means to match the two bilingual groups. The results of the additional tasks describing the characteristics of the participants (the language history questionnaire) and measuring their language proficiency (the lexical decision task and the semantic verbal fluency task) and cognitive abilities/resources (the Simon task and the operation span task) are summarized in Table 1.

#### Materials

##### Pictures

Sixteen black-and-white line drawings were sampled from Snodgrass and Vanderwart (1980), Székely et al. (2003) and Székely et al. (2004) based on the following criteria: 1) all pictures had noncognate names in English, Spanish, and Japanese; 2) all pictures were typically written in kanji for Japanese^1^; 3) the phonological onset of the English name of pictures was restricted to phonology that was shared with Spanish and Japanese.^2^ In addition to the experimental pictures, eight filler pictures that met the first two criteria were included in the present experiment. None of the fillers was the same as the experimental pictures.

##### Distractor Words

For each of the pictures, four types of distractor words were selected in Spanish: phonologically related to the English picture name, semantically related to the English picture name, Spanish translation name of the English picture name, phonologically related to the Spanish translation name of the English picture name (phono-translation) (see Table 2 for examples). The following criteria were used to select each type of distractor: 1) distractors that were phonologically related to the English picture name or to the Spanish translation of the English picture name were matched on phonological onset with the English picture name and were not also semantically related to the target picture; 2) semantically related distractors were largely from the same semantic categories and were not phonologically related to the English or Spanish name of the picture. Each of these related distractors had an unrelated control that was matched item-by-item based on length (number of characters and syllables) and frequency [all *p*s > 0.10] (see Table 2). Because it was not possible to match distractors across distractor types completely without weakening the relation of the pair within each condition, we analyzed the data for each distractor type separately. Each of the filler pictures also had a distractor word in Spanish. The complete set of the experimental items is provided (Supplementary Appendix A).

In the present study, each experimental picture was presented eight times but with different distractors so that none of the distractor words was repeated. Likewise, filler pictures were also repeated eight times. Unlike the experimental trials, however, distractors for filler pictures were each presented four times. Each list had eight blocks and each of the eight blocks included 16 experimental pictures and eight filler pictures. There were two items for each type of distractor and each of its unrelated controls per block for the experimental pictures. Each block started with two filler trials and the critical trials and the rest of the filler trials were presented randomly within the block. The order of blocks was counterbalanced across participants.

#### Procedure

Participants first received written instructions in English on the computer screen. They were informed that a series of pictures would be presented with an L1 (Spanish) distractor word one at a time on the computer screen. Their task was to name the pictured object in English as quickly and accurately as possible while ignoring the L1 distractor word. At the beginning of each trial, a fixation sign (+) was presented at the center of the computer screen. At the press of a button, the fixation sign was replaced with a blank screen and 500 ms after the offset of the fixation sign, a picture was presented. A distractor word appeared in red at the center of the picture 25 ms after the onset of the presentation of the picture. The picture and the distractor word were presented until the participants responded or for 5,000 ms. If they did not know the name of the object, they were told to say “no”. After they responded, a blank screen was presented for 500 ms and a fixation sign appeared again. The 25 ms delay was included to ensure that participants could first see the pictured object clearly. Eight practice trials were presented twice prior to the experimental trials. The pictures and distractors used in the practice trials were different from the experimental items.

#### Data Trimming Procedure

Recorded picture naming responses were first transcribed and coded for accuracy. We included only the expected picture names as correct responses in order to maintain the phonological manipulation. Responses that deviated from the expected picture name, responses that started with an article or hesitation, and “no” responses were scored as errors (5.6%). Responses that the microphone did not detect were eliminated as technical errors (<0.1%). Correct responses that were less than 300 ms or greater than 2,500 ms were identified as outliers (1.1%) and excluded from the analyses.

#### Data Analysis

Statistical analyses were performed using linear and generalized mixed-effects models (Baayen et al., 2008) in the lme4 software package (v. 1.1.23; Bates et al., 2015) in the R statistical software environment (v. 4.0.2; R Core Team, 2020). For picture naming latency analyses, models had log response times (RTs) as the dependent variable and distractor type (related or unrelated) as a dummy-coded fixed effect. For picture naming accuracy, we ran logistic mixed-effects regression with accuracy as the dependent variable and distractor type (related vs. unrelated) as a dummy-coded fixed effect. To guard against Type I errors and increase generalizability (Barr et al., 2013), we attempted to fit random effects including both random intercepts and random slopes. However, these models returned a warning message for singularity (i.e., where one or more variances are estimated as zero). To allow a non-singular fit, random slopes were removed and thus the random effect structure for both RT and accuracy models contained only random intercepts for participants and items.^3^

### Results

**FIGURE 1 |:**
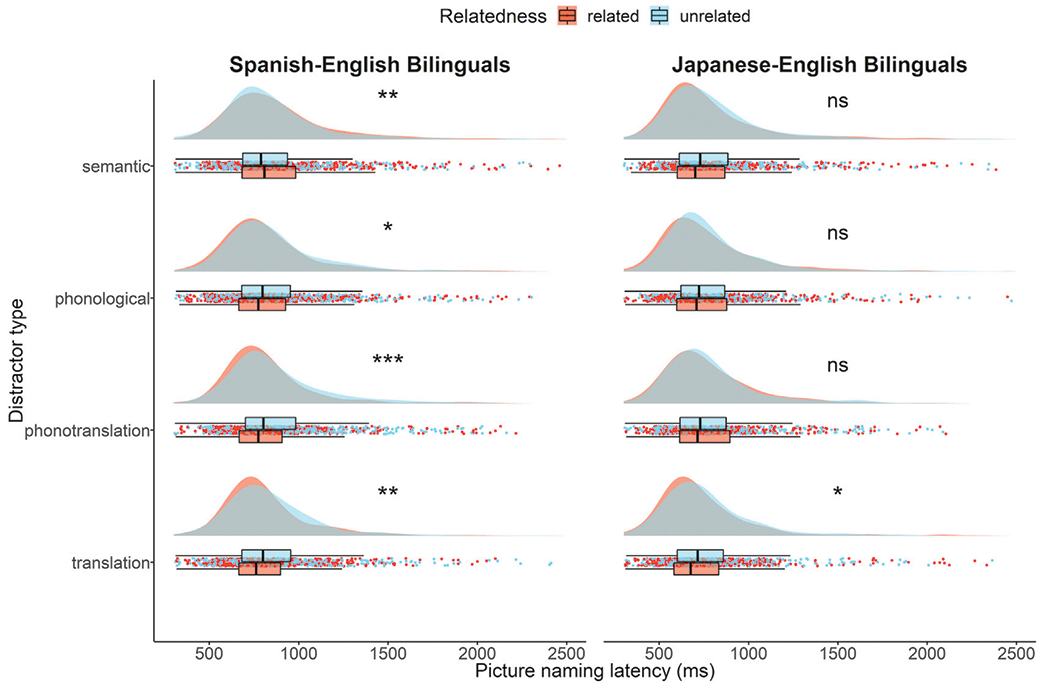
Raincloud plots (Allen et al., 2019) showing distributions, boxplots, and raw data of picture naming latencies (in milliseconds) for related and unrelated trials across semantic, phonological, phono-translation, and translation distractor types for Spanish-English **(left)** and Japanese-English **(right)** bilingual groups. Statistical analyses were performed on log transformed response times. Significance codes: ns = not significant, *p < 0.05, **p < 0.01, ***p < 0.001.

Table 3 shows mean picture naming latencies (ms) and accuracy (percent correct) across the four distractor types. Naming latencies associated with related and unrelated conditions are additionally shown in Figure 1. Full model outputs are provided (Supplementary Appendix C).

Spanish-English bilinguals named pictures more slowly (*β* = −0.02, SE = 0.01, *t* = −2.90, *p* = 0.004, 95% CI [−0.03 to −0.01]) and less accurately (*β* = 0.67, SE = 0.29, *z* = 2.34, *p* = 0.019,95% CI [0.11–1.24]) when distractor words were semantically related than when they were semantically unrelated. Furthermore, relative to unrelated controls, participants named pictures faster when distractor words were phonologically related (*β* = 0.02, SE = 0.01, *t* = 2.46, *p* = 0.014, 95% CI [0.00–0.03]), phonologically related to Spanish translations of English picture names (*β* = 0.02, SE = 0.01, *t* = 3.81, *p* < 0.001, 95% CI [0.01–0.04]), or Spanish translations of English picture names (*β* = 0.02, SE = 0.01, *t* = 2.61, *p* = 0.009, 95% CI [0.00–0.03]). Picture naming accuracy did not differ between related and unrelated conditions for phonological (*β* = 0.08, SE = 0.28, *z* = 0.30, *p* = 0.763, 95% CI [−0.46–0.63]), phono-translation (*β* = −0.04, SE = 0.28, *z* = −0.14, *p* = 0.889, 95% CI [−0.58–0.51]) or translation (*β* = −0.20, SE = 0.26, *z* = −0.76, *p* = 0.445, 95% CI [−0.70–0.31]) distractor types.

### Discussion

In Experiment 1, Spanish-English bilinguals showed the effect of all the distractor types—phonological facilitation, semantic interference, translation facilitation, and phono-translation facilitation. In other words, we replicated the general pattern of results reported for previous bilingual picture-word interference studies with bilinguals whose two languages share the same Roman alphabets. This replication was found despite the fact that the Spanish-English bilinguals in Experiment 1 did not share the same language profile with respect to age of L2 acquisition, language environment, and language proficiency with the bilinguals tested in the previously published studies (e.g., Hermans et al., 1998; Costa and Caramazza, 1999; Costa et al., 1999; Hermans, 2000; Hermans, 2004; Costa et al., 2003; Knupsky and Amrhein, 2007).

Although we replicated a general pattern of the results of previous bilingual picture-word interference studies for Spanish-English bilinguals, one issue that requires additional discussion concerns the phono-translation effect. The phono-translation distractor words in Experiment 1 for same-script Spanish-English bilinguals produced facilitation rather than interference. Hermans et al. (1998) found an effect of interference for phono-translation distractors for Dutch-English bilinguals. A critical difference between their study and the present study was in the stimulus construction. In Hermans et al., the distractor words were phonologically related, semantically related, phono-translation, or unrelated, whereas the present study included translation distractors in addition to those four types of distractors. When translation names of pictures are included in the task, phono-translation distractors appear to facilitate picture naming rather than interfere the process of speech planning. In other words, just as translation distractor words facilitate the selection of target pictures, phono-translation distractor words activate translation names of pictures and make lexical selection easier. In another study, we did find, like Hermans et al., that Spanish-English bilinguals living in an L2 English environment showed phono-translation interference rather than facilitation when the task did not include translation distractors (Hoshino and Thierry, 2011). This interpretation is also consistent with the results of Hermans et al. (2011) showing that the presence of cross-language activation in a phoneme monitoring task was sensitive to the composition of the experimental materials. Only when there were cognates present in the list context, was cross-language activation observed.

In Experiment 2, we asked whether different-script Japanese-English bilinguals would also show the same pattern of the results as same-script Spanish-English bilinguals in Experiment 1. In the absence of a language cue, Spanish-English and Japanese-English bilinguals perform similarly on a simple picture naming task (Hoshino and Kroll, 2008). If Japanese distractors words provide a language cue to the language of production, then Japanese-English bilinguals should show smaller cross-language effects than Spanish-English bilinguals.

## EXPERIMENT 2: JAPANESE-ENGLISH BILINGUALS

### Method

#### Participants

Thirty-nine Japanese-English bilinguals who were living in the L2 environment at the time of testing participated in Experiment 2. They completed the same set of tasks as Spanish-English bilinguals in Experiment 1. The characteristics of the Japanese-English bilinguals are summarized in Table 1.

#### Materials

##### Pictures

The pictures were identical to those used in Experiment 1.

##### Distractor Words

For each of the pictures, four types of distractor words were selected in Japanese: phonologically related to the English picture name, semantically related to the English picture name, Japanese translation name of the English picture name, phonologically related to the Japanese translation name of the English picture name (phono-translation) (see Table 4 for examples). Similar to Experiment 1, the following criteria were used to select each type of distractor: 1) the distractors that were phonologically related to the English picture name or to the Japanese translation of the English picture name were matched on phonological onset with the English picture name and were not also semantically related to the target picture; 2) words were typically written in kanji; 4) the semantically related distractors were identical to those in Experiment 1 (i.e., the English translation of the semantically related distractors were the same). Each of these related distractors had an unrelated control that was matched item-by-item based on length (number of characters and syllables) and frequency [all *p*s > 0.10] (see Table 4). Each of the filler pictures also had a distractor word in Japanese. The complete set of the experimental items is provided (Supplementary Appendix B). The organization of the lists and blocks was identical to the one in Experiment 1.

##### Norming

Although phonologically related and phono-translation distractors were matched on phonological onset with English picture names in both Experiment 1 and Experiment 2, an additional measure of phonological similarity of the distractors and picture names was obtained to ensure that observed cross-language differences between Experiment 1 and Experiment 2, if any, were due to script but not to differences in phonological similarity. Fifteen English monolinguals who had not studied Spanish and 16 English monolinguals who had not studied Japanese were asked to rate sound pairs according to how similar two words sounded on a 7-point Likert scale with “1” being completely different and “7” being identical.^4^ The sound pairs consisted of an English picture name and its phonologically related or unrelated Spanish/Japanese distractor word and pairs consisting of a Spanish/Japanese picture name and its phonologically related or unrelated Spanish/Japanese distractor word. The English picture names were recorded by a female native speaker of English and the Spanish and Japanese distractor words were recorded by female native speakers of Spanish and Japanese, respectively. The sound file of each distractor word in Spanish or in Japanese was combined with that of the target English picture name or the target Spanish/Japanese picture name. A set of English-Spanish/Japanese sound pairs and a set of Spanish-Spanish or Japanese-Japanese sound pairs were created. Each stimulus set consisted of 32 sound pairs and therefore, each participant received 64 sound pairs.

The mean ratings for each condition are summarized by sound pairs in Table 5. A critical result in the norming experiment was that monolingual English speakers perceived the phonological similarity of related pairs to be greater than unrelated pairs in the phonological condition [*t*(15) = 7.61, *p* < 0.001 for English-Spanish pairs; *t*(15) = 11.25, *p* < 0.001 for English-Japanese pairs] and in the phono-translation condition [*t*(15) = 17.68, *p* < 0.001 for Spanish-Spanish pairs; *t*(15) = 14.31, *p* < 0.001 for Japanese-Japanese pairs], regardless of language pairs. Although care was taken to ensure that phonological similarity would be similar across experiments, the English names of pictures and their phonologically related Japanese distractors were rated as more similar than those of the English names of pictures and their phonologically related Spanish distractors [*t*(15) = 3.12, *p* < 0.01]. However, it is important to note that if this difference in phonological similarity of items influences bilingual performance, then Japanese-English bilinguals in Experiment 2 should show greater phonological facilitation than Spanish-English bilinguals in Experiment 1, which would counter the predicted reduction of distractor effects for different-script bilinguals.

#### Procedure

The same procedure was used as in Experiment 1 except for that the distractor words were Japanese, not Spanish.

#### Data Trimming Procedure

The data trimming procedure was identical to Experiment 1. Errors (4.8%), technical errors (<0.1%), and outliers (1.0%) were excluded from the data analyses.

#### Data Analysis

The same procedure was used as in Experiment 1.

### Results

Table 3 shows mean picture naming latencies (ms) and accuracy (percent correct) across the four distractor types. Naming latencies associated with related and unrelated conditions are additionally shown in Figure 1. Full model outputs are provided (Supplementary Appendix C).

In contrast to Spanish-English bilinguals, Japanese-English bilinguals did not show a semantic interference effect (naming latency: (*β* = 0.00, SE = 0.01, *t* = 0.29, *p* = 0.772, 95% CI [−0.01–0.02]); naming accuracy: *β* = 0.21, SE = 0.33, *z* = 0.65, *p* = 0.516, 95% CI [−0.43–0.85]). They also did not show relatedness effects in naming latency or accuracy for phonological (naming latency: *β* = 0.01, SE = 0.01, *t* = 1.21, *p* = 0.228, 95% CI [−0.01–0.02]; naming accuracy: *β* = −0.07, SE = 0.30, *z* = −0.22, *p* = 0.826, 95% CI −0.65–0.52]) or phono-translation (naming latency: *β* = 0.00, SE = 0.01, *t* = 0.26, *p* = 0.796, 95% CI [−0.01–0.02]; naming accuracy: *β* = −0.16, SE = 0.32, *z* = −0.48, *p* = 0.628, 95% CI [−0.79–0.48]) distractor types. However, Japanese-English bilinguals named pictures faster when distractor words were Japanese translations of English picture names than when they were unrelated (*β* = 0.02, SE = 0.01, *t* = 2.29, *p* = 0.022, 95% CI [0.00–0.03]), but naming accuracy did not differ in this condition (*β* = −0.25, SE = 0.32, *z* = −0.79, *p* = 0.431, 95% CI [−0.87–0.37]).

### Discussion

Unlike findings for simple picture naming, in which the performance of Spanish-English and Japanese-English bilinguals was identical (Hoshino and Kroll, 2008), Spanish-English bilinguals (Experiment 1) and Japanese-English bilinguals (Experiment 2) performed differently on picture naming in the presence of language-specific distractor words. Similar to Spanish-English bilinguals in Experiment 1, Japanese-English bilinguals showed translation facilitation. Unlike Spanish-English bilinguals, however, they did not show phonological facilitation, semantic interference, and phono-translation facilitation. The absence of phonological and phono-translation effects might be due to the characteristics of kanji scripts. Past research suggests that phonology is specified earlier in kana than in kanji, whereas semantic access occurs earlier in kanji than in kana (e.g., Yamada, 1998; Ischebeck, 2004; Chen et al., 2007). A critical finding in the present study is that Japanese-English bilinguals did not show semantic interference even with kanji distractor words, and this finding is consistent with the results of the picture-word interference studies with bimodal bilinguals (Giezen and Emmorey, 2016; Emmorey et al., 2020b). In sum, these results suggest that when the distinctive script is present in the task, different-script bilinguals are able to exploit the perceptual information as a cue to allow language selection to occur earlier in speech planning relative to same-script bilinguals.

## GENERAL DISCUSSION

The goal of the present study was to determine whether the degree of cross-language activation and the locus of language selection could be modulated by script when the task included an overt written lexical form. Spanish-English bilinguals (Experiment 1) and Japanese-English bilinguals (Experiment 2) named pictures in their L2 English while ignoring visually presented L1 (Spanish/Japanese) distractor words. The distractor words were manipulated in relation to the picture to create four conditions: phonological, semantic, translation, and phono-translation. Unlike findings for simple picture naming, Spanish-English and Japanese-English bilinguals in the present study performed differently on picture naming in the presence of language-specific distractor words. In the picture-word interference task, both groups showed translation facilitation, whereas only Spanish-English bilinguals demonstrated phonological facilitation, semantic interference, and phono-translation facilitation. In other words, we replicated a general pattern of the results of previous bilingual picture-word interference studies for same-script Spanish-English bilinguals, whereas the pattern of results differed for different-script Japanese-English bilinguals.

We now consider why Japanese-English bilinguals showed translation facilitation, but not phonological facilitation, semantic interference, and phono-translation facilitation. We argue that when the distinctive script is present in the task, different-script bilinguals are able to exploit the perceptual information as a cue to allow language selection to occur earlier in speech planning relative to same-script bilinguals. According to this account, lexical candidates from both languages are activated for a very brief period of time but speech planning then becomes language-selective such that only lexical candidates from the target language (i.e., English) compete for selection. As can be seen in Table 3, Japanese-English bilinguals were faster to name pictures than Spanish-English bilinguals although the two groups were matched on verbal fluency, which was a measure of productive skills, and if anything, Spanish-English bilinguals appeared more proficient in English on other measures. This difference might also reflect the early language selection by Japanese-English bilinguals.^5^ Indeed, this account is in line with studies showing that Chinese-English bilinguals named images culturally matched with the language to be spoken faster than those culturally mismatched (e.g., Jared et al., 2013; Li et al., 2013), that bimodal bilinguals did not show semantic interference in naming pictures in American Sign Language (ASL) while ignoring English distractor words (Giezen and Emmorey, 2016; Emmorey et al., 2020a), and that Hebrew-English bilinguals read aloud mixed-language texts more accurately than Spanish-English bilinguals (Fadlon et al., 2019). This is also compatible with studies showing that different-script bilinguals coactivate the non-target language in a semantic relatedness judgment task where the non-target language is only implicitly available (Thierry and Wu, 2007; Degani et al., 2018).

On this account of “early selection”, distractors are unlikely to have an effect except when they are the translation of the picture name because the phonological and semantic representation of the distractor will be available only after language selection has occurred. Why would there be an effect for translation equivalents but not for other semantic related distractors? It appears that the semantic activation of the picture itself primed the recognition of the distractor when it was the translation, i.e., the name of the picture, to create convergence among related conceptual nodes. There are two results in the past literature that suggest that resonance among activated lexical codes may be a critical factor. Previous studies of bilingual word recognition (e.g., Dijkstra et al., 1998) have shown that cognates produce more robust cross-language effects than interlingual homographs. The analogy with the present result is that semantic conflicts across languages can sometimes be ignored whereas semantic convergence can almost never be ignored (see Schwartz and Kroll, 2006, for an illustration of the same phenomenon within sentence context). Another feature of the translation facilitation effect that is relevant to the present discussion is that in experiments in which the SOA has been manipulated between the presentation of the picture and distractor, translation facilitation only occurs very early. Because the data for Japanese-English bilinguals suggest that they are not able to selectively ignore the Japanese distractor words until some processing of the distractor has occurred, those processes that reflect early interactions between the bottom-up activation of the word and the top-down information engaged by the picture, are likely to survive the cross-language script difference. In Emmorey et al. (2020a), in fact, ASL-English bilinguals showed the effect of relatedness in the time window of 200–300 ms for the translation condition but not for the semantic condition.

In the present study, both the Spanish-English and the Japanese-English bilinguals produced translation facilitation, whereas only the Spanish-English bilinguals produced phonological facilitation, semantic interference, and phono-translation facilitation. The pattern of these results suggests that script differences modulate cross-language activation during production when the written lexical form is perceptually available in one language. The distinctive script appears to serve as a language cue to direct attention to the lemmas in the target language alone at an earlier stage of speech planning, a finding that is in line with the assumption that the flow of activation is nonselective but the manner of language selection may be language-specific (e.g., Costa and Caramazza, 1999; Costa et al., 1999). In other words, even if there is cross-language activation, the activation of lexical candidates from the non-response language does not necessarily interfere with lexical selection in the intended language if there is a basis on which the language of speaking can be selected in advance. However, the fact that the presence of the script difference alone was not sufficient to create an entirely selective, monolingual-like situation for the Japanese-English bilinguals, is compatible with a model that assumes that all activated lexical candidates from both languages compete for selection (e.g., Green, 1998). Different scripts may function to inhibit unintended alternatives earlier in the process, thereby eliminating phonological facilitation, semantic interference, and phono-translation facilitation, but they cannot override cross-language activation entirely.

Alternatively, the Response Exclusion Hypothesis assumes that competition occurs at a post-lexical level, not at a lexical level (Mahon et al., 2007). On this account, distractor words activate their representations in the articulators prior to the picture. Because the articulators are a single-channel buffer, non-target representations need to be excluded to articulate the target picture name. When more features are shared between the target picture name and distractors, it takes longer to reject non-target candidates from the buffer. The absence of the distractor effects other than the translation facilitation for Japanese-English bilinguals may be due to the fact that it takes longer to have access to the phonological properties of kanji distractor words. Unlike alphabetic writing systems, the pronunciation of a kanji character is not transparent because its components do not correspond to the individual phonemes of the pronunciation, which makes it take longer to retrieve the phonology of the character. By the time kanji distractors activate the representations in the articulators, the language of production is already selected.

Another possible account is based on a model that assumes that lexical alternatives in the non-target language are further from the selection criteria (threshold) and thus are rejected more easily than alternatives in the response language (Finkbeiner et al., 2006). This threshold model posits that the bilingual’s intention to speak in one language activates the target language more strongly than the non-target language and lexical candidates in the target language will reach the threshold for selection more quickly. The absence of phonological, semantic, and phono-translation effects for Japanese-English bilinguals might be explained by the threshold account if we assume that the distinctive script does not meet the selection criterion and lexical alternatives in the nonresponse language can be rejected rapidly. However, if the absence of phonological, semantic, and phono-translation effects were due to the adjustment of selection criteria, then translation facilitation should also have not been obtained. It is important to note that in the present study, the distractor conditions were mixed so that the Japanese-English bilinguals could not simply set a different threshold strategically, depending upon the type of distractor words.

In summary, the present study replicated a general pattern of the results of past bilingual picture-word interference studies for same-script bilinguals (Spanish-English) but only partly for different-script bilinguals (Japanese-English). This specific pattern of the present results suggests that when script is perceptually available, the degree of cross-language activation and the locus of language selection is modulated by script differences between the bilingual’s two languages. Based on the obtained results, we have argued that the flow of activation in the mechanism of language production is fundamentally nonselective. Language-specific differences such as script can serve as a language cue to allow the bilingual to select the intended language earlier in the process of speech planning when they are perceptually available. That is, these findings suggest that the locus of language selection in bilingual speech planning is “not” fixed (see Kroll et al., 2006 for a review). The fact that some, but not all, distractor conditions were effective for the Japanese-English bilinguals is consistent with an account of bilingual production in which activated candidates in the non-target languages are suppressed earlier in speech planning when language status is available. If different-script bilinguals had been better able to attend to the target language from the start, then no effects of the distractors should have been observed. In future research, it will be critical to further examine the time course of language/lexical selection as a function of type of bilingualism (same script vs. different script).

## Supplementary Material

Hoshino et al. (2021) - SI

## Figures and Tables

**TABLE 1 |:** Comparisons of Spanish-English (Experiment 1) and Japanese-English bilinguals (Experiment 2) on proficiency and cognitive measures.

Measure	Spanish-English	Japanese-English	*p*
	(Experiment 1)	(Experiment 2)	
Age (years)	25.7 (5.9)	27.2 (7.7)	0.32
L1 self-rating (10 pt scale)	9.4 (0.8)	9.1 (1.1)	0.11
L2 self-rating (10 pt scale)	8.5 (1.3)	7.0 (1.8)	<0.001
Daily L1 usage (%)	43.2 (25.1)	39.5 (26.8)	0.51
Daily L2 usage (%)	57.9 (23.8)	57.8 (26.2)	0.98
Age of L2 acquisition (years)	8.3 (4.8)	10.6 (3.7)	0.01
Length of immersion (months)	87.4 (78.1)	79.1 (72.1)	0.62
Lexical decision			
RT for nonword(ms)	937 (260)	1,017 (301)	0.18
RT for word (ms)	683 (112)	729 (177)	0.17
Accuracy for nonword (%)	83.3 (11.6)	78.8 (10.8)	0.07
Accuracy for word (%)	94.1 (3.7)	93.5 (3.0)	0.44
Verbal fluency in L1^a^	12.2 (2.9)	11.8 (1.7)	0.40
Verbal fluency in L2^a^	11.1 (2.0)	10.8 (2.0)	0.36
Simon effect (ms)^b^	46.7 (33.4)	28.9 (22.2)	0.01
RT for neutral (ms)	466 (96)	404 (52)	<0.001
RT for congruent (ms)	446 (97)	390 (58)	0.002
RT for incongruent (ms)	493 (94)	419 (52)	<0.001
Accuracy for neutral (%)	99.4 (1.6)	98.8 (2.0)	0.18
Accuracy for congruent (%)	99.1 (1.7)	98.5 (2.1)	0.16
Accuracy for incongruent (%)	96.7 (4.2)	97.0 (3.4)	0.74
Operation span (0–60)^c^	33.9 (10.5)	39.2 (7.4)	0.01
RT for equation judgment (ms)	2537 (235)	2182 (251)	<0.001
Errors for equation judgment (0–60)	14.6 (8.3)	8.6 (4.7)	<0.001

Standard deviations are in parentheses.

a The verbal fluency score is the mean exemplars per category.

b The Simon effect is the difference in RTs between congruent and incongruent conditions.

c The operation span is the number of words that were recalled correctly among correct responses to the equation judgment, which were not considered as outliers.

**TABLE 2 |:** Examples of distractors for the picture “envelope” by distractor type and relatedness and characteristics of Spanish distractors.

Distractor type	Examples		Frequency		Length	
	Related	Unrelated	Related	Unrelated	Related	Unrelated
Phonological	enchufe (plug)	rodilla (knee)	2.015	2.004	5.4 (2.3)	5.6 (2.4)
Semantic	tarjeta (postcard)	alicates (pliers)	1.735	1.819	5.8 (2.4)	6.1 (2.6)
Translation	sobre (envelope)	hombre (man)	2.061	2.065	5.2 (2.2)	5.3 (2.2)
Phono-translation	sobrino (nephew)	paloma (pigeon)	1.614	1.603	5.8 (2.6)	5.8 (2.6)

(1) The translation of the distractor word is given in parentheses.

(2) The number of letters is provided without parentheses and the number of syllables is provided with parentheses.

Frequency was from Alameda and Cuetos (1995) and log frequency was computed based on the values from the source.

**TABLE 3 |:** Picture naming mean latencies and accuracy (standard deviation in parenthesis) for related and unrelated trials in Experiment 1 and Experiment 2.

Distractor type	Experiment 1		Experiment 2	
	Spanish-English bilinguals		Japanese-English bilinguals	
	Related	Unrelated	Related	Unrelated
Latency (ms)				
Semantic	881 (305)	844 (275)	794 (308)	789 (273)
Phonological	834 (285)	861 (281)	781 (285)	789 (276)
Phono-translation	831 (273)	879 (287)	786 (270)	793 (277)
Translation	819 (253)	852 (277)	748 (284)	772 (269)
Accuracy (% correct)				
Semantic	93.8 (2.4)	96.0 (2.0)	94.5 (2.3)	95.2 (2.1)
Phonological	94.9 (2.2)	95.2 (2.1)	94.5 (2.3)	94.3 (2.3)
Phono-translation	95.1 (2.2)	94.9 (2.2)	95.4 (2.1)	94.8 (2.2)
Translation	94.7 (2.2)	93.9 (2.4)	95.2 (2.1)	94.3 (2.3)

**TABLE 4 |:** Examples of distractors for the picture “envelope” by distractor type and relatedness and characteristics of Japanese distractors.

Distractor type	Examples		Frequency		Length	
	Related	Unrelated	Related	Unrelated	Related	Unrelated
Phonological	煙突 (chimney)/eNtotu/	大根 (radish)/daikoN/	3.375	3.390	1.7 (2.7)	1.7 (2.7)
Semantic	葉書 (postcard)/hagaki/	毛虫 (caterpillar)/kemusi/	3.409	3.390	1.5 (2.7)	1.5 (2.7)
Translation	封筒 (envelope)/huRtoR/	花火 (firework)/hanabi/	3.642	3.390	1.3 (2.8)	1.3 (2.7)
Phono-translation	風鈴 (wind-bell)/huRriN/	王冠 (crown)/oRkaN/	3.670	3.390	1.7 (2.7)	1.7 (2.7)

(1) The translation of the distractor word and its phonemic transcription are given in parentheses and in slashes, respectively.

(2) The number of characters is provided without parentheses and the number of morae is provided with parentheses.

Frequency was from Amano and Kondo (2000) and log frequency was computed based on the values from the source.

**TABLE 5 |:** Similarity ratings by English monolinguals as a function of distractor type and relatedness.

Distractor type	Spanish		Japanese	
	Related	Unrelated	Related	Unrelated
Phonological	3.6 (1.3)	1.2 (0.2)	4.6 (1.3)	1.3 (0.2)
Phono-translation	4.1 (0.5)	1.4 (0.5)	4.3 (0.7)	1.4 (0.3)

Standard deviations are in parentheses.

## Data Availability

The original contributions presented in the study are included in the article/Supplementary Material. Further inquiries can be directed to the corresponding author.

## Footnotes

1 We included the criteria (2) and (3) to use an identical set of pictures for Experiments 1 and 2. In Japanese, some words can be written both in kanji and in kana (hiragana and katakana), whereas others can be written only in kana (hiragana and katakana). Although some words can be written in either writing system, there are preferences for one rather than the other. The preference was determined by the frequency of each form (Amano and Kondo, 2000).

2 For example, “frog” was not included according to the third criterion because the consonant cluster/fr/was not possible in Japanese. Although these criteria limited the number of items in the present experiment, it was critical to use these criteria to make the contribution of phonologically related distractors equivalent for Spanish-English and Japanese-English bilinguals.

3 Per the editor’s suggestion, we conducted additional analyses including phonological similarity ratings and cognitive measures as predictors, but we found that none of these contributed to model fit. Thus, they were not included in the final models.

4 Two independent groups of English monolinguals were recruited to minimize the effect of speakers.

5 Alternatively, the absence of phonological, semantic, and phono-translation effects for Japanese-English bilinguals might have been due to individual differences in the ability to ignore irrelevant information and in the availability of the amount of processing resources. As shown in Table 1, the Japanese-English bilinguals produced a smaller Simon effect and a larger operation span than the Spanish-English bilinguals. These cognitive measures did not improve model fit in both Experiment 1 and Experiment 2. However, it is critical to investigate the extent to which cognitive control abilities as well as script differences can contribute to cross-language distractor effects in future research.
