# Supplementary material for: Do Cross-Language Script Differences Enable Bilinguals to Function Selectively When Speaking in One Language Alone?
Source: Front Commun (Lausanne). Author manuscript; Available in PMC 2022 Apr 12. (PMC9004719; doi:10.3389/fcomm.2021.668381)
Supplement: Hoshino et al. (2021) - SI [file NIHMS1790614-supplement-Hoshino_et_al___2021__-_SI.pdf]

Appendix A

Pictures and Spanish distractor words.

Picture	Phonological	Phonological control	Semantic	Semantic control	Translation	Translation control	Phono-translation	Phono-translation control
car	casa	mundo	tren	copa	coche	pelo	colegio	hermana
chicken	chico	cuello	cisne	limón	pollo	blusa	pozo	mono
cow	causa	autor	caballo	dientes	vaca	gorra	vaho	hoyo
desk	descanso	frontera	estante	cuchara	escritorio	bicicleta	escalera	bolsillo
dog	doctor	botella	gato	baño	perro	carta	percha	payaso
envelope	enchufe	alicates	tarjeta	rodilla	sobre	hombre	sobrino	paloma
mirror	mira	río	ventana	corazón	espejo	labios	espada	anillo
sock	saco	piano	guante	mosca	calcetín	acordeón	calendario	escarabajo
balloon	valle	isla	pelota	maleta	globo	casco	gloria	cuento
bell	vela	león	iglesia	teléfono	campana	chaleco	camisa	piedra
book	vuelta	cocina	revista	montaña	libro	calle	lirio	chapa
bone	voto	lana	músculo	caracol	hueso	cañón	huevo	ducha
box	valor	seguro	lata	pera	caja	dedo	cama	dinero
eye	aire	paso	nariz	reloj	ojo	pan	ojal	piña
king	quinta	pulgar	soldado	bandera	rey	piel	repollo	barril
sun	zanahoria	trompeta	luna	radio	sol	cara	sofá	hilo

Appendix B

Pictures and Japanese distractor words (with phonemic transcriptions).

Picture	Phonological	Phonological control	Semantic	Semantic control	Translation	Translation control	Phono-translation	Phono-translation control
car	蚊 /ka/	酢 /su/	電車 /deNsja/	児童 /zidoR/	車 /kuruma/	額 /hitai/	雲 /kumo/	旗 /hata/
chicken	地球 /tikjuR/	舞台 /butai/	白鳥 /hakutjoR/	大砲 /taihoR/	鶏 /niwatori/	唇 /kutibiru/	庭 /niwa/	板 /ita/
cow	顔 /kao/	水 /mizu/	豚 /buta/	松 /matu/	牛 /usi/	腹 /hara/	海 /umi/	山 /jama/
desk	出口 /deguti/	指輪 /yubiwa/	棚 /tana/	琴 /koto/	机 /tukue/	涙 /namida/	津波 /tunami/	汽船 /kiseN/
dog	道具 /doRro/	戦車 /seNsja/	猫 /neko/	城 /siro/	犬 /inu/	髪 /kami/	意図 /ito/	文字 /mozi/
envelope	煙突 /entotu/	大根 /daikoN/	葉書 /hagaki/	毛虫 /kemusi/	封筒 /huRtoR/	花火 /hanabi/	風鈴 /huRriN/	王冠 /oRkaN/
mirror	未来 /mirai/	神社 /ziNzja/	窓 /mado/	脳 /noR/	鏡 /kagami/	桜 /sakura/	科学 /kagaku/	料理 /rjoRri/
sock	作家 /saQka/	教師 /kjoRsi/	手袋 /tebukuro/	天使 /teNsi/	靴下 /kutusita/	戦艦 /seNkaN/	苦痛 /kutuR/	切手 /kiQte/
balloon	罰 /batu/	滝 /taki/	球 /tama/	影 /kage/	風船 /fuRseN/	親指 /ojajubi/	夫婦 /huRhu/	手紙 /tegami/
bell	別荘 /beQsoR/	真実 /siNzitu/	教会 /kjoRkai/	人形 /niNgjoR/	鐘 /kane/	舌 /sita/	加熱 /kanetu/	倉庫 /soRko/
book	部下 /buka/	屋根 /jane/	雑誌 /zaQsi/	投手 /toRsju/	本 /hoN/	家 /ie/	保護 /hogo/	維持 /izi/
bone	盆地 /boNti/	定規 /zjoRgi/	筋肉 /kiNniku/	鉛筆 /eNpitu/	骨 /hone/	雪 /juki/	保険 /hokeN/	市長 /sitjoR/
box	幕府 /bakuhu/	気質 /kisitu/	缶 /kaN/	糸 /ito/	箱 /hako/	馬 /uma/	墓 /haka/	敵 /teki/
eye	愛 /ai/	空 /sora/	鼻 /hana/	皿 /sara/	目 /me/	地 /ti/	綿 /meN/	菊 /kiku/
king	金庫 /kiNko/	辞書 /zisjo/	兵士 /heisi/	時計 /tokeR/	王 /oR/	口 /kuti/	応募 /oRbo/	秘密 /himitu/
sun	酸 /saN/	沼 /numa/	月 /tuki/	花 /hana/	太陽 /taijoR/	警官 /keikaN/	体重 /taizjuR/	教訓 /kyoRkuN/

Appendix C

Model Summaries
Picture Naming Latency

Spanish-English Bilinguals: Semantic Distractor
Formula: RT_log ~ Relatedness + (1 | ParticipantID) + (1 | PictureID)
Data: spa_rt_sem
Control: lmerControl(optimizer = "bobyqa")
REML criterion at convergence: -1828.1

Scaled residuals:

Min	1Q	Median	3Q	Max
-3.7356	-0.5669	-0.0989	0.4336	4.4741

Random effects:

Groups	Name	Variance	Std.Dev.
ParticipantID	(Intercept)	0.003579	0.05982
PictureID	(Intercept)	0.000411	0.02027
Residual		0.014812	0.12171

Number of obs: 1427, groups: ParticipantID, 48; PictureID, 16
Fixed effects:

	Estimate	Std. Error	df	t value	Pr(>|t|)
(Intercept)	2.929082	0.01102	61.57502	265.8087	6.2E-96
Relatednessunrelated	-0.01971	0.006448	1364.002	-3.05658	0.002282

Spanish-English Bilinguals: Phonological Distractor
Formula: RT_log ~ Relatedness + (1 | ParticipantID) + (1 | PictureID)
Data: spa_rt_phono
Control: lmerControl(optimizer = "bobyqa")
REML criterion at convergence: -1933.7

Scaled residuals:

Min	1Q	Median	3Q	Max
-3.3785	-0.5997	-0.1262	0.4546	4.5226

Random effects:

Groups	Name	Variance	Std.Dev.
ParticipantID	(Intercept)	0.0033316	0.05772
PictureID	(Intercept)	0.0003333	0.01826
Residual		0.0137679	0.11734

Number of obs: 1427, groups: ParticipantID, 48; PictureID, 16
Fixed effects:

	Estimate	Std. Error	df	t value	Pr(>|t|)
(Intercept)	2.902318	0.010479	62.17278	276.9718	7.87E-98
Relatednessunrelated	0.015381	0.006215	1364.04	2.474736	0.013454

Spanish-English Bilinguals: Phonotranslation Distractor
Formula: RT_log ~ Relatedness + (1 | ParticipantID) + (1 | PictureID)
Data: spa_rt_phonotrans
Control: lmerControl(optimizer = "bobyqa")
REML criterion at convergence: -1997.3

Scaled residuals:

Min	1Q	Median	3Q	Max
-3.6813	-0.5616	-0.1513	0.4579	3.9646

Random effects:

Groups	Name	Variance	Std.Dev.
ParticipantID	(Intercept)	0.0024364	0.04936
PictureID	(Intercept)	0.0005571	0.02360
Residual		0.0131981	0.11488

Number of obs: 1426, groups: ParticipantID, 48; PictureID, 16
Fixed effects:

	Estimate	Std. Error	df	t value	Pr(>|t|)
(Intercept)	2.903096	0.010219	53.24347	284.0971	2.26E-86
Relatednessunrelated	0.023111	0.006088	1363.148	3.795915	0.000154

Spanish-English Bilinguals: Translation Distractor
Formula: RT_log ~ Relatedness + (1 | ParticipantID) + (1 | PictureID)
Data: spa_rt_trans
Control: lmerControl(optimizer = "bobyqa")
REML criterion at convergence: -2033.8

Scaled residuals:

Min	1Q	Median	3Q	Max
-4.0229	-0.5655	-0.1136	0.4408	4.8905

Random effects:

Groups	Name	Variance	Std.Dev.
ParticipantID	(Intercept)	0.0022681	0.04762
PictureID	(Intercept)	0.0006862	0.02619
Residual		0.0126737	0.11258

Number of obs: 1413, groups: ParticipantID, 48; PictureID, 16
Fixed effects:

	Estimate	Std. Error	df	t value	Pr(>|t|)
(Intercept)	2.896114	0.010397	47.84293	278.5569	1.84E-78
Relatednessunrelated	0.01623	0.005994	1350.473	2.707496	0.006865

Japanese-English Bilinguals: Semantic Distractor
Formula: RT_log ~ Relatedness + (1 | ParticipantID) + (1 | PictureID)
Data: jap_rt_sem
Control: lmerControl(optimizer = "bobyqa")
REML criterion at convergence: -1594.2

Scaled residuals:

Min	1Q	Median	3Q	Max
-3.2983	-0.5807	-0.1365	0.4858	4.5187

Random effects:

Groups	Name	Variance	Std.Dev.
ParticipantID	(Intercept)	0.0053338	0.07303
PictureID	(Intercept)	0.0003571	0.01890
Residual		0.0133561	0.11557

Number of obs: 1166, groups: ParticipantID, 39; PictureID, 16
Fixed effects:

	Estimate	Std. Error	df	t value	Pr(>|t|)
(Intercept)	2.876469	0.013502	50.76089	213.0365	1.34E-76
Relatednessunrelated	0.005186	0.006774	1111.668	0.76551	0.44413

Japanese-English Bilinguals: Phonological Distractor
Formula: RT_log ~ Relatedness + (1 | ParticipantID) + (1 | PictureID)
Data: jap_rt_phono
Control: lmerControl(optimizer = "bobyqa")
REML criterion at convergence: -1614.1

Scaled residuals:

Min	1Q	Median	3Q	Max
-3.8790	-0.5806	-0.1354	0.4868	4.0622

Random effects:

Groups	Name	Variance	Std.Dev.
ParticipantID	(Intercept)	0.0048686	0.06978
PictureID	(Intercept)	0.0006186	0.02487
Residual		0.0130885	0.11441

Number of obs: 1166, groups: ParticipantID, 39; PictureID, 16
Fixed effects:

	Estimate	Std. Error	df	t value	Pr(>|t|)
(Intercept)	2.873801	0.013643	52.29149	210.6432	2.85E-78
Relatednessunrelated	0.008881	0.006706	1110.91	1.32421	0.185706

Japanese-English Bilinguals: Phonotranslation Distractor
Formula: RT_log ~ Relatedness + (1 | ParticipantID) + (1 | PictureID)
Data: jap_rt_phonotrans
Control: lmerControl(optimizer = "bobyqa")
REML criterion at convergence: -1653.7

Scaled residuals:

Min	1Q	Median	3Q	Max
-3.8600	-0.5856	-0.1398	0.4806	4.8071

Random effects:

Groups	Name	Variance	Std.Dev.
ParticipantID	(Intercept)	0.0052020	0.07212
PictureID	(Intercept)	0.0003218	0.01794
Residual		0.0127532	0.11293

Number of obs: 1170, groups: ParticipantID, 39; PictureID, 16
Fixed effects:

	Estimate	Std. Error	df	t value	Pr(>|t|)
(Intercept)	2.878161	0.013243	49.6443	217.3423	1.29E-75
Relatednessunrelated	0.002887	0.006608	1114.34	0.436932	0.662246

Japanese-English Bilinguals: Translation Distractor
Formula: RT_log ~ Relatedness + (1 | ParticipantID) + (1 | PictureID)
Data: jap_rt_trans
Control: lmerControl(optimizer = "bobyqa")
REML criterion at convergence: -1571.8

Scaled residuals:

Min	1Q	Median	3Q	Max
-3.1562	-0.5515	-0.1124	0.4746	4.5832

Random effects:

Groups	Name	Variance	Std.Dev.
ParticipantID	(Intercept)	0.0054730	0.07398
PictureID	(Intercept)	0.0005054	0.02248
Residual		0.0136743	0.11694

Number of obs: 1172, groups: ParticipantID, 39; PictureID, 16
Fixed effects:

	Estimate	Std. Error	df	t value	Pr(>|t|)
(Intercept)	2.857023	0.013982	51.69878	204.3363	7.58E-77
Relatednessunrelated	0.015329	0.006836	1117.391	2.242517	0.025123

Picture Naming Accuracy

Spanish-English Bilinguals: Semantic Distractor

Family: binomial (logit)
Formula: Accuracy ~ Relatedness + (1 | ParticipantID) + (1 | PictureID)
Data: spa_acc_sem
Control: glmerControl(optimizer = "bobyqa", optCtrl = list(maxfun = 100000))

AIC	BIC	logLik	deviance	df.resid
478.5	499.9	-235.3	470.5	153

Scaled residuals:

Min	1Q	Median	3Q	Max
-7.5230	0.0521	0.0842	0.1551	2.0441

Random effects:

Groups	Name	Variance	Std.Dev.
ParticipantID	(Intercept)	1.913	1.383
PictureID	(Intercept)	3.448	1.857

Number of obs: 1535, groups: ParticipantID, 48; PictureID, 16
Fixed effects:

	Estimate	Std. Error	z value	Pr(>|z|)
(Intercept)	4.513962	0.636194	7.095256	1.29E-12
Relatednessunrelated	0.629439	0.277517	2.268112	0.023322

Spanish-English Bilinguals: Phonological Distractor

Family: binomial (logit)
Formula: Accuracy ~ Relatedness + (1 | ParticipantID) + (1 | PictureID)
Data: spa_acc_phono
Control: glmerControl(optimizer = "bobyqa", optCtrl = list(maxfun = 100000))

AIC	BIC	logLik	deviance	df.resid
472.7	494.0	-232.3	464.7	1530

Scaled residuals:

Min	1Q	Median	3Q	Max
-13.1945	0.0512	0.0830	0.1702	1.3885

Random effects:

Groups	Name	Variance	Std.Dev.
ParticipantID	(Intercept)	1.453	1.206
PictureID	(Intercept)	4.334	2.082

Number of obs: 1534, groups: ParticipantID, 48; PictureID, 16
Fixed effects:

	Estimate	Std. Error	z value	Pr(>|z|)
(Intercept)	4.877702	0.721444	6.761023	1.37E-11
Relatednessunrelated	0.079579	0.271273	0.293355	0.76925

Spanish-English Bilinguals: Phonotranslation Distractor

Family: binomial (logit)
Formula: Accuracy ~ Relatedness + (1 | ParticipantID) + (1 | PictureID)
Data: spa_acc_phonotrans
Control: glmerControl(optimizer = "bobyqa", optCtrl = list(maxfun = 100000))

AIC	BIC	logLik	deviance	df.resid
469.9	491.3	-231.0	461.9	1532

Scaled residuals:

Min	1Q	Median	3Q	Max
-12.9337	0.0525	0.0817	0.1656	1.3456

Random effects:

Groups	Name	Variance	Std.Dev.
ParticipantID	(Intercept)	1.025	1.012
PictureID	(Intercept)	4.359	2.088

Number of obs: 1536, groups: ParticipantID, 48; PictureID, 16
Fixed effects:

	Estimate	Std. Error	z value	Pr(>|z|)
(Intercept)	4.855831	0.711185	6.827803	8.62E-12
Relatednessunrelated	-0.03616	0.268899	-0.13447	0.893027

Spanish-English Bilinguals: Translation Distractor

Family: binomial (logit)
Formula: Accuracy ~ Relatedness + (1 | ParticipantID) + (1 | PictureID)
Data: spa_acc_trans
Control: glmerControl(optimizer = "bobyqa", optCtrl = list(maxfun = 100000))

AIC	BIC	logLik	deviance	df.resid
553.1	574.4	-272.5	545.1	1532

Scaled residuals:

Min	1Q	Median	3Q	Max
-11.1112	0.0873	0.1248	0.1873	1.2476

Random effects:

Groups	Name	Variance	Std.Dev.
ParticipantID	(Intercept)		
PictureID	(Intercept)		

Number of obs: 1536, groups: ParticipantID, 48; PictureID, 16
Fixed effects:

	Estimate	Std. Error	z value	Pr(>|z|)
(Intercept)	4.08127	0.472284	8.64156	5.55E-18
Relatednessunrelated	-0.1827	0.24718	-0.73913	0.459829

Japanese-English Bilinguals: Semantic Distractor

Family: binomial (logit)
Formula: Accuracy ~ Relatedness + (1 | ParticipantID) + (1 | PictureID)
Data: jap_acc_sem
Control: glmerControl(optimizer = "bobyqa", optCtrl = list(maxfun = 100000))

AIC	BIC	logLik	deviance	df.resid
388.2	408.7	-190.1	380.2	1244

Scaled residuals:

Min	1Q	Median	3Q	Max
-10.6232	0.0790	0.1124	0.1664	0.9762

Random effects:

Groups	Name	Variance	Std.Dev.
ParticipantID	(Intercept)	0.5871	0.7662
PictureID	(Intercept)	2.5490	1.5966

Number of obs: 1248, groups: ParticipantID, 39; PictureID, 16
Fixed effects:

	Estimate	Std. Error	z value	Pr(>|z|)
(Intercept)	4.166316	0.54967	7.579671	3.46E-14
Relatednessunrelated	0.221459	0.298381	0.742203	0.457964

Japanese-English Bilinguals: Phonological Distractor

Family: binomial (logit)
Formula: Accuracy ~ Relatedness + (1 | ParticipantID) + (1 | PictureID)
Data: jap_acc_phono
Control: glmerControl(optimizer = "bobyqa", optCtrl = list(maxfun = 100000))

AIC	BIC	logLik	deviance	df.resid
410.0	430.5	-201.0	402.0	1243

Scaled residuals:

Min	1Q	Median	3Q	Max
-9.2403	0.0890	0.1235	0.1766	1.0194

Random effects:

Groups	Name	Variance	Std.Dev.
ParticipantID	(Intercept)	0.3875	0.6225
PictureID	(Intercept)	2.3011	1.5170

Number of obs: 1247, groups: ParticipantID, 39; PictureID, 16
Fixed effects:

	Estimate	Std. Error	z value	Pr(>|z|)
(Intercept)	4.076709	0.515909	7.901996	2.74E-15
Relatednessunrelated	-0.06036	0.287077	-0.21025	0.833476

Japanese-English Bilinguals: Phonotranslation Distractor

Family: binomial (logit)
Formula: Accuracy ~ Relatedness + (1 | ParticipantID) + (1 | PictureID)
Data: jap_acc_phonotrans
Control: glmerControl(optimizer = "bobyqa", optCtrl = list(maxfun = 100000))

AIC	BIC	logLik	deviance	df.resid
347.1	367.7	-169.6	339.1	1244

Scaled residuals:

Min	1Q	Median	3Q	Max
-10.1827	0.0571	0.0874	0.1526	1.0434

Random effects:

Groups	Name	Variance	Std.Dev.
ParticipantID	(Intercept)	0.2974	0.5454
PictureID	(Intercept)	4.0283	2.0071

Number of obs: 1248, groups: ParticipantID, 39; PictureID, 16
Fixed effects:

	Estimate	Std. Error	z value	Pr(>|z|)
(Intercept)	4.831202	0.726028	6.654289	2.85E-11
Relatednessunrelated	-0.14488	0.311066	-0.46576	0.641388

Japanese-English Bilinguals: Translation Distractor

Family: binomial (logit)
Formula: Accuracy ~ Relatedness + (1 | ParticipantID) + (1 | PictureID)
Data: jap_acc_trans
Control: glmerControl(optimizer = "bobyqa", optCtrl = list(maxfun = 100000))

AIC	BIC	logLik	deviance	df.resid
400.4	420.9	-196.2	392.4	1244

Scaled residuals:

Min	1Q	Median	3Q	Max
-9.1369	0.0841	0.1269	0.1742	1.2834

Random effects:

Groups	Name	Variance	Std.Dev.
ParticipantID	(Intercept)	0.4494	0.6704
PictureID	(Intercept)	2.3917	1.5465

Number of obs: 1248, groups: ParticipantID, 39; PictureID, 16
Fixed effects:

	Estimate	Std. Error	z value	Pr(>|z|)
(Intercept)	4.231202	0.534122	7.921795	2.34E-15
Relatednessunrelated	-0.21288	0.292497	-0.72779	0.466743
